# Body surface area capping may not improve cytotoxic drugs tolerance

**DOI:** 10.1038/s41598-021-81792-6

**Published:** 2021-01-28

**Authors:** Wafa Bouleftour, Agathe Viard, Benoite Mery, Robin Chaux, Nicolas Magne, Xavier Simoens, Romain Rivoirard, Fabien Forges

**Affiliations:** 1 Medical Oncology Department, Lucien Neuwirth Cancer Center, 42270 Saint Priest en Jarez, France; 2 Pharmacy Department, Lucien Neuwirth Cancer Center, 42270 Saint-Priest en Jarez, France; 3 Clinical Research, Innovation and Pharmacology Unit, University Hospital of Saint-Etienne, 42000 Saint Etienne, France; 4 Radiotherapy Department, Lucien Neuwirth Cancer Center, 42270 Saint Priest en Jarez, France

**Keywords:** Cancer, Medical research

## Abstract

Capping body surface area (BSA) at 2 m^2^ is a routine clinical practice. It aims at reducing toxicities in over 2 m^2^ BSA patients. 455,502 computerized chemotherapy prescriptions made between 2011 and 2017 were taken from BPC software. Chemotherapy computerized order entry is created by a senior physician prescribers before patient consultation. Only prescriptions with dose calculation involving BSA were selected. 51,179 chemotherapy prescriptions were analyzed; corresponding to 7206 patients who received intravenous chemotherapy. The number of chemotherapy prescriptions in over 2 m^2^ BSA patients was nearly the same in the hematology as in the oncology departments. But, 79.1% of prescriptions were capped at 2 m^2^ in the oncology department contrary to 21.9% in the hematology department. Practices analysis showed more dose limitation in palliative situations in both departments. Unexpectedly, 6.53% of capped prescriptions were performed in patients with normal BMI. The patients who received capped doses of chemotherapy had neither fewer dose reductions due to toxicity nor deterioration of their general condition. Capping did not induce fewer dose reductions in patients with BSA greater than 2 m^2^. Prospective studies in this population are needed to standardize chemotherapy administration in population with BSA > 2 m^2^.

## Introduction

What characterizes injectable anticancer drugs is their narrow therapeutic index, posology liked with dose intensity and significant variable non-specific toxic effects. In an attempt to maximize anti-tumor effects associated with a good tolerance of treatment and acceptable levels of toxicity, drugs prescriptions were based on body surface area (BSA). Historically, BSA has been the dose calculation indicator that best limits inter-individual variations^1^. Indeed, BSA allows to normalize some physiological parameters influencing pharmacokinetic (cardiac output, left ventricular mass, renal clearance) and to determine the dose of drug to be administered in patients with different body sizes^2,3^. These parameters have been shown to be better correlated with BSA than other weight descriptors^4–6^. BSA formula was originally developed by Du bois and Du bois^7^. This formula dates back to 1916, it was validated on nine patients whose weight ranged from 25 to 90 kg. It was not designed for obese or underweight population. Many other formulae have been developed to sharpen BSA estimation but unfortunately they have resulted in a too high variability between formulae^8^. None of the formulae gives acceptable estimation for unstandardized populations regarding fat and lean body mass, especially in cancer patients who are often over or underweight, or presenting edema. Given pharmacokinetic parameters uncertainty such as volume of distribution or clearances, overweight patients were usually considered at risk of overdosing. In order to limit toxic effects, other more suitable BSA estimators were tested^9,10^ such as the use of ideal body weight^11^, limitation of BSA to 2 m^2^, reduction of doses. These dose reductions may partially explain poor clinical outcomes in the obese population^12^.

Capping BSA at 2 m^2^ is a priori a common clinical practice used by physicians to reduce toxicities in over 2 m^2^ BSA patients. The traditional use of this empiric practice could be explained by the fear of overdosing and misestimating BSA in patients with extreme height and weight. Yet, this practice seems to be mainly related to obesity as extreme BSA is largely linked with overweight.

Nevertheless, such medical practice is supported by neither scientific rational nor clinical studies. It is an arbitrary practice, which does not take into account body mass index (BMI): tall patients with normal BMI are usually capped and certainly underdosed. Moreover, short obese patients with BSA less than 2 m^2^ are not considered as at risk of overdosing. BSA estimation could lead to substantial underestimations and thus, to weak antitumor activity^8^. Indeed, the formula Du bois and Du bois mostly used would already underestimate BSA^13^. Moreover, capping BSA arbitrarily is not pharmacologically satisfying given the heterogeneity of pharmacokinetic parameters for each drug. In addition, there is no pharmacokinetic arguments to support this practice. Even if few clinical trials decree capping, there is no oncological guideline. All clinical trials focused on capping BSA practice in obese populations. ASCO guidelines recommend against chemotherapy dose reduction in obese patients, in order to avoid compromising clinical outcomes^12^. Moreover, ASCO reported that more than 40% of obese patients received adjusted chemotherapy doses without any justification^12^.

Therefore, given to the scarcity of data, more studies are needed to define a consensus of capping practice use. The aim of this study was to describe practices regarding chemotherapy doses capping within a French anticancer hospital. Thereby, risk factors influencing capping practices and the correlation between capping and toxicities were analyzed.

## Results

### Characteristics of patients receiving intravenous chemotherapy prescription

Between January 1st, 2011 and the May 31st, 2017, 7206 patients received intravenous chemotherapy prescription. The sex ratio (M/W) in this population was 0.8 (56.8% of women). 64% of this population had a BMI below 25 kg/m^2^ while 24.5% and 11.5% of patients were respectively overweight (BMI 25–29.9) and obese (BMI ≥ 30). 8.3% of patients had a BSA greater than 2 m^2^. All patient characteristics are summarized in Table 1.

**Table 1:** Patients characteristics (n = 7206).

Variable	Patients receiving intravenous chemotherapy (n = 7206)
Age (years)	
Median [Q1–Q3]	64 [54–72]
Gender	
Men (%)	3113 (43.2%)
Women (%)	4093 (56.8%)
BMI (kg/m^2^)	
Underweight (< 18.5)	763 (10.5%)
Normal weight (18.5–24.9)	3859 (53.5%)
Overweight (25–29.9)	1762 (24.5%)
Obesity (30–34.9)	822 (11.5%)
BSA (m^2^)	
> 2 m^2^	595 (8.3%)
≤ 2 m^2^	6611 (91.7%)

### Global analysis of chemotherapy prescriptions

7206 patients received 51,179 chemotherapy prescription. 41,773 (81.6%) were prescribed within the medical oncology department and 9406 (18.4%) within the hematology department.

For solid cancer treatments, 25.1%, 22.5%, 8.8% and 7.3% of chemotherapy prescriptions were for digestive, breast, gynecological and lung cancer, respectively. Lymphoma, acute leukemia and myeloma received 9.8%, 2.5% and 2.3% of all prescriptions. (Table 2).

**Table 2:** Distribution of prescriptions by cancer location.

Department	Localization	N	%
Medical oncology	Digestive	12,862	25.1
	Breast	11,513	22.5
	Gynecological	4526	8.8
	Lung	3717	7.3
	Prostate	2023	4
	UADC	2883	2.6
	Urological	1343	2.6
	CNS	1228	2.4
	Sarcoma	1103	2.2
	Other	486	0.9
	Dermatological	80	0.2
Hematology	Lymphoma	5022	9.8
	Acute Leukemia	1279	2.5
	Myeloma	1197	2.3
	CLL	852	1.7
	Myelodysplasic syndrome	532	1
	Graft	503	1
	Other	21	0.1
Total		51,179	100

*UADC* upper aero digestive cancer, *CNS* central nervous system, *CLL* chronic lymphocytic leukemia.

### Distribution of chemotherapy prescriptions according to medical indication and body surface area

Out of 51,179 chemotherapy prescriptions, the percentage of chemotherapy prescriptions for patients with BSA over 2 m^2^ was nearly the same in the oncology department as in the hematology department. Indeed, these prescriptions accounted for respectively 10% and 15% of the total number of prescriptions in both departments (Table 3). Interestingly in the population with BSA > 2 m^2^, the sex ratio (M/W) is 4.46 (18.3% of women with BSA > 2 m^2^. Data not shown).

**Table 3:** Chemotherapy prescriptions according to medical indication and body surface area.

Department	BSA ≤ 2 m^2^		BSA > 2 m2	
	N	%	N	%
Medical oncology (N = 41,773)	37,493	89.75	4280	10.25
BSA > 2				
m^2^ (N = 4280)				
Capped doses	–	–	3386	79.1
Uncapped doses	–	–	894	20.9
Hematology (N = 9406)	7951	84.53	1455	15.47
BSA > 2				
m^2^ (N = 1455)				
Capped doses	–	–	319	21.9
Uncapped	–	–	1136	78

5735 chemotherapy prescriptions were administered in patients with BSA > 2 m^2^. Respectively, 79.1% and 21.9% of chemotherapy prescription were capped to 2 m^2^ in oncology and hematology departments. (Table 3). Interestingly, capping approach depended on the practice of each physician. It could be divided into 3 groups: rare capping (less than 30%), variable capping (30–70%) and systematic capping (more than 70%) (data not shown).

### Distribution of chemotherapy prescriptions according to BMI and body surface area

Out of the 51,179 chemotherapy prescriptions, 11.21% were for patients with BSA > 2 m^2^. More precisely, 1.18% (n = 606), 4.48% (n = 2292), 5.54% (n = 2836) of these prescriptions were dispensed respectively in patients with normal, overweight and obese BMI. (Table 4). Interestingly, 0.73% (n = 375) of the patients who received capped chemotherapy doses, had normal BMI. It corresponded to 6.53% of capped prescriptions.

**Table 4:** Distribution of prescriptions according to IMC, and body surface (SC ≤ 2 m^2^ and SC > 2 m^2^).

BMI (kg/m^2^) (N = 51,179)	BSA ≤ 2 m^2^		BSA > 2 m^2^	
	N%		N%	
**Underweight (< 18.5)**	3655	7.14	1	0.002
Capped	–		0	0
Uncapped	–		1	0.002
**Normal weight (18.5–24.9)**	25,326	49.49	606	1.18
Capped	–		375	0,73
Uncapped	–		231	0,45
**Overweight (25–29.9)**	12,036	23.52	2292	4.48
Capped	–		1518	2.96
Uncapped	–		774	1.52
**Obese (≥ 30)**	4427	8.65	2836	5.54
Capped	–		1812	3.54
Uncapped	–		1024	2
Total	45,444	88.79	5735	11.21

### Intent of treatment and capping

In the medical oncology department, the therapeutic objective was curative for 35.8% of capped prescriptions, and for 47.3% of uncapped doses, whereas in the hematology department, the curative approach was observed for 94.3% of capped prescriptions and 97.6% of uncapped doses. (Table 5). Curative approach consisted of adjuvant and neoadjuvant treatment for solid tumors, and allogenic/autologous stem cell transplantation, consolidation, intensification, maintenance, and induction for hematological cancers.

**Table 5:** Therapeutic approach and chemotherapy dose limitation.

Intent of treatment	Department	Capped prescription		Uncapped prescription	
		N	%	N	%
Curative	Medical oncology	1212	74.1	423	25.6
	Hematology	300	21.4	1104	78.6
	Sub-total	1512	49.8	1527	50.2
Palliative	Medical oncology	2174	81.2	471	17.8
	Hematology	18	40	27	60
	Sub-total	2192	81.5	498	18.5

Practices analysis showed more dose limitations in palliative situations in both departments (oncology: 74% vs 82, hematology: 21% vs 40%). This difference was clearer in the hematology department.

### Dose reduction in prescriptions with BSA > 2 m^2^

No difference was observed in dose reduction between capped and uncapped prescription. Indeed, dose reduction was observed respectively in 17.7% and 18.86% of capped and uncapped prescriptions. (Table 6A). As first cycle dose selection is a crucial parameter, data base analysis showed no difference in dose reduction at 1st cycle between patient with less or over 2 m^2^ BSA (13.68% vs 12.98%) (Data not shown).

**Table 6:** (A) Dose reduction in prescriptions with BSA > 2 m^2^, (B) Dose reduction induced by BSA capping according to the intent of treatment.

(A) Dose reduction	Capped prescription (N = 3705)		Uncapped prescription (N = 2030)	
	N	%	N	%
Yes	631	17.7	383	18.86
No	3074	82.3	1647	81.14

(B) Intent of treatment	Dose reduction		N	%
Curative	0–3%		654	43.25
	3–20‚%		849	56.15
	> 20		9	0.60
	Sub-total		1512	100
Palliative	0–3%		843	38.46
	3–20%		1341	61.18
	> 20		8	0.36
	Sub-total		2192	100

In population with BSA > 2 m^2^, capping was observed in 40.8% in curative approach and in 59.1% in palliative care (Table 6B). Dose reduction induced by BSA capping was arbitrarily considered insignificant when it was less than 3%. This threshold is usually applied by the pharmacy department during the production of injectable chemotherapies.

In curative treatment, BSA capping induced a significant dose reduction in 56.7% of prescription. Similarly, 36.4% of significant dose reduction were observed in palliative care.

### Logistic regression analysis

Multivariate logistic regression model showed that age [OR: 1.016; *P* = 0.02], sex [OR: 2.01; *p* = 0.01] and type of tumor [OR: 27.24; *p* < 0.001] are significant risk factors explaining the use of the capping method. Interestingly, palliative intent of treatment was only significant in univariate analysis [OR: 1.58; *p* = 0.005]. BMI was not significant [OR: 0.98; *p* = 1]. (Table 7A).

**Table 7:** Univariate and multivariate logistic regression (A) capping as dependent variable, (B) dose reduction as dependent variable.

	Univariate		Multivariate	
	OR [95% CI]	*p*	OR [95% CI]	*p*
**(A) Logistic regression model (dependent variable :capping)**				
**Age**	1.01 [1.00–1.02]	**0.0595**	1.016 [1.005–1.028]	**0.02945**
**Sex**				
Female	1.83 [1.22–2.74]	**0.017**	2.01 [1.25–3.23]	**0.0193**
Male	*ref*		*ref*	
**BMI**	1.01 [0.99–1.04]	1	0.9897 [0.9626–1.0177]	1
**Localization**				
Liquid	*ref*		*ref*	
Solid	26.14 [16.23–80.45]	**< 0.001**	27.24 [11.80–62.87]	**< 0.001**
**Curative/Palliative**				
Curative	*ref*		*ref*	
Palliative	1.58 [1.20–2.08]	**0.0055**	1.42 [1.05–1.90]	0.1051
**(B) Logistic regression model (dependent variable : dose reduction)**				
**Age**	1.09 [1.06–1.13]	**< 0.001**	1.07 [1.05–1.10]	**< 0.001**
**Curative/palliative**				
Curative	*ref*		*ref*	
Palliative	3.68 [2.33–5.79]	**< 0.001**	2.98 [1.90–4.67]	**< 0.001**
**Capping**				
Yes	1.13 [0.81–1.57]	1	1.06 [0.77–1.45]	1
No	*ref*		*ref*	

Age [OR: 1.07; *p* < 0.001] and palliative intent of treatment [OR: 2.98; *p* < 0.001] are significant discriminating factors to explain dose reduction related to toxicities and/or deterioration of general condition. Capping was not a significant factor related to dose reduction [OR: 1.06; *p* = 1] (Table 7B).

## Discussion

The lack of consensus regarding the proper use of injectable neoplastic drugs in patients with BSA > 2 m^2^ is a major issue. In 2017, a questionnaire filled by French oncologists to assess practices regarding BSA capping (unpublished data). Physicians declared they always (9%), often (18%), rarely (27%), never (46%) limited BSA to 2 m^2^. They were mostly unaware of the formula they used to estimate BSA. Indeed, prescribing practice analysis showed that oncologists capped more often injectable chemotherapy doses than hematologists. This could be explained by the fact that in hematology, the intent of treatment is more often curative than in oncology. Moreover, drugs and associated dosages used in oncology are not the same as in hematology, which induced a distinct tolerance profile.

BMI analysis of our population, showed that 0.73% of patients -corresponding to 6.53% of capped prescriptions- had normal BMI. Indeed, a man measuring 198 cm for 71.7 kg has a BMI of 18.3 kg/m^2^ and a BSA estimated at 2.05 m^2^ according to the Du Bois and Du Bois formula. Although BSA and BMI depend on the same anthropometric characteristics (height and weight), there is no need to conflate high BSA with obesity. Thus, patients with BSA strictly greater than 2 m^2^ may therefore be underweight or have a normal, overweight or obese BMI. Indeed, patients with normal BMI and a BSA > 2 m^2^, in whom pharmacokinetic parameters are not altered should never undergo “capping” chemotherapy doses. This empirical practice can induce a loss of luck in these patients unfairly receiving capped doses of chemotherapy. Furthermore, computer tomography-based body surface area evaluation gives a promising results in drug dosage^14^. With high precision and accuracy, this method provide a sensitive measurement and could be a research area in chemotherapy methods of calculation.

**Figure 1:**
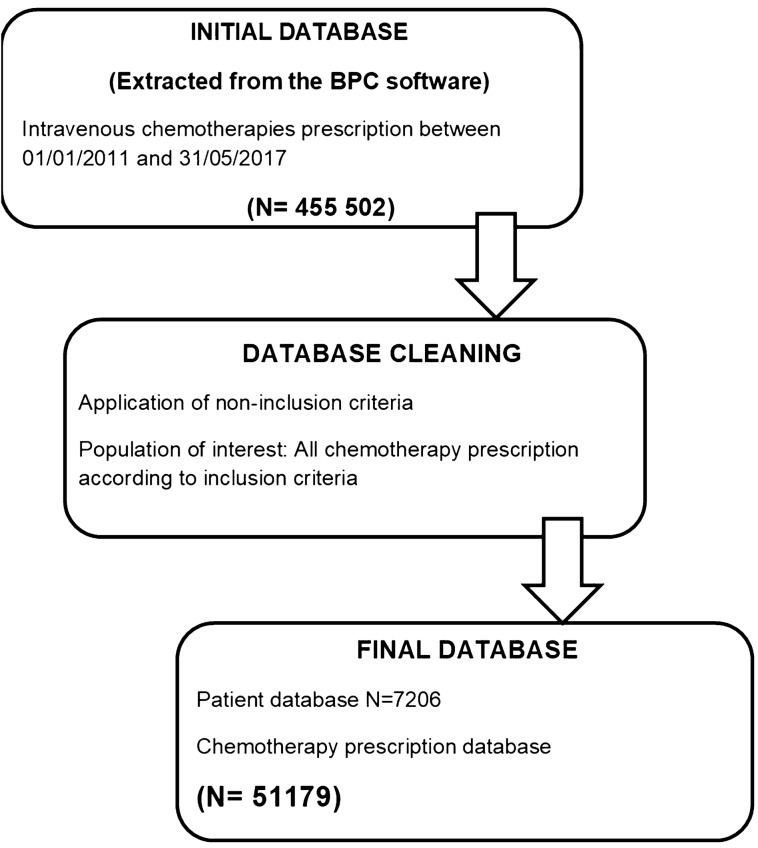
Schematic plan of the methodology of data collection.

In this study, practice analysis showed more dose limitations were decided in palliative situations in both oncology and hematology departments. Indeed, in curative situations, the administration of a full chemotherapy dose is essential in order to optimize treatment efficiency. The full dose administered can be considered as an indicator of quality of care^12^. Furthermore, capping practices did not change over the time. Indeed, after the introduction in 2012 of the ASCO clinical practice guidelines recommending full weight-based dosing, conversely there was a tendency to capping practices increase within our center (59% in 2013, 77% in 2014, 64% in 2017; Fig. 1.supp material).

As expected, chemotherapy prescriptions analysis showed that capping increased with age, type of tumor (solid) and sex (female) whereas BMI and the intent of treatment had no impact. Age, sex and palliative situations are probably correlated. These results therefore suggest that the practice of capping depends mainly on the therapeutic situation and not on anthropometric criteria. This is also supported by the lack of dose adjustment for obese patients with BSA < 2 m^2^. Regression results also showed female are twice more likely to be capped. This could be explained by the fact that the median age of death in female cancer patients is 73 vs 77 in male. In addition, breast cancer is the most common cancer in women worldwide. The reference treatment is curative adjuvant chemotherapy. Conversely, patients with prostate cancer requiring chemotherapy treatment are mainly metastatic. (In the study chemotherapy, prescription were for breast cancer (22, 5%) and prostate cancer (4%). See Table 2).

Capping did not impact dose reduction related to toxicity or deterioration in general condition in patients with BSA greater than 2 m^2^. In other words, the practice of capping a priori does not seem to reduce treatment toxicities.In accordance, several retrospective studies performed in obese patients with breast, colon or ovarian cancers, concluded that capping did not induce higher risk of toxicities^15–20^, and recommended a full chemotherapy doses in obese population. Conversely, GAIN study observed an increase in toxicities in 31% of obese patients receiving chemotherapy after an unadjusted BSA. 69% of patients enrolled in this study received chemotherapy doses on basis of an adjusted BSA (97% of BSA was adjusted to an ideal weight and 3% were capped to 2 m2)^21^.

This retrospective study analyzed more than 51,000 chemotherapy prescriptions. The main limitation of this study are firstly that BPC does not allow to export the data relating to a dose adjustment at 0%. Thus, treatment interruptions due to toxicity were not taken into account. Secondly, we could not conclude in this work if the capping is intentional or made by mistake. Indeed, ergonomics specific to the BPC software could induce an unintentional limitation if the physician did not select the right option either by ignorance of the software or by negligence.

In conclusion, despite all limitations of retrospective studies, this work showed that unexpectedly 6.53% of capped prescriptions were performed in patients with normal BMI. Furthermore, capping did not induce more dose reduction in over 2m^2^ BSA patients. Bouleftour et al. review highlighted the lack of prospective studies focusing on chemotherapy methods of administration in obese patients^22^. Indeed, based on the results of this work, prospective clinical trials are crucial in order to bring scientific proof to this empirical practice. Moreover, pharmacological studies are needed to find a consensus and a reliable formula for patients whose BSA is over 2. Finally, the exploitation of computerized tomography images for the determination of BSA evaluation should be validated through prospective clinical trials.

## Material and methods

### Data acquisition

This retrospective observational study analyzed all intravenous chemotherapy prescriptions between January 1st 2011 and May 31st 2017 made in Lucien Neuwirth Cancer Center (Saint Priest en Jarez, France). This hospital includes all onco-hematology specialties except pediatric oncology. This study was approved by Saint Etienne local ethics committee. Furthermore, given to the nature of the study—electronic database analysis—Saint Etienne local ethics committee waived the need of the informed consent. All methods were performed in accordance with the relevant guidelines and regulations of retrospective studies. Thus, the aim of this study was to analyze risk factors influencing capping chemotherapy prescriptions. The correlation between capping and dose reductions was also analyzed.

The prescriptions were taken from the prescription software BPC, implemented in the hospital since 2011. Chemotherapy computerized order entry is created by a senior physician prescribers before patient consultation, and then it’s validated following the medical consultation by the doctor who consults the patient. 55 502 computerized prescription were taken in EXCEL format. A total of 51,179 intravenous chemotherapy prescriptions were then selected (Fig. 1).

The inclusion criteria were intravenous chemotherapy prescriptions with a dose calculation using BSA. After data extraction, many parameters were already available (weight, height, BSA, age, dose reduction, therapeutic line, type of cancer). Some were added such as BMI, BSA limitation, curative or palliative intent of treatment. All dose reductions were motivated either by chemotherapy toxicities or a deterioration of patient's general condition. For example, systematic dose capping at 2 mg for vincristine was not considered as a dose reduction. Furthermore, prescriptions to patients included in a clinical trial were excluded from the analysis because of the frequent instructions for dose calculation. Protocols requiring dose escalation were also excluded.

The BPC software uses the Du Bois and Du Bois formula to calculate patients’ BSA. In case of BSA superior to 2 m^2^, physicians are asked by the software to choose between capping or not.

Baseline demographics information of patients receiving intravenous chemotherapy prescription were collected. The age was calculated based on the date of the chemotherapy prescription and the date of birth of the patient. The BMI (Body Mass Index) was calculated according to the weight and height of the patient at the time of prescription. According to BMI score patients were classified in four groups: BMI < 18.5 for underweight patient; BMI between 18.5 and 24.9 for normal patient; BMI between 25 and 29.9 for overweight patient; and BMI > 30 for obese patient.

### Statistical and data analysis

All variables collected were described using the following methods: median (standard deviation), for quantitative variables; size (percentage) for qualitative variables.

Analysis were performed using univariate and multivariate mixed effect logistic regression models. Models were constructed a priori, with inclusion of potential confounders and variables of interest based on prior knowledge on the topic and clinical relevance.

To identify independent risk factors for capping, a multivariate mixed effect regression model was used, with age, BMI, cancer localization and curative or palliative approach as fixed effects. The model was further adjusted for patients and prescribing physician as random effects.

To identify independent risk factors for dose reduction induced by toxicities or an impaired general condition, a multivariate mixed effect regression model was used, with age, curative or palliative approach and capping as fixed effects. The model was further adjusted for patients and prescribing physician as random effects.

Prior to both multivariate analysis, correlations and interactions were systematically assessed between variables of interest.

All tests were two sided and a *p* value < 0.05 was considered to be statistically significant. To account of multiple testing, the *p* values were also adjusted using Bonferroni correction. Statistical analyzes were performed using the R language and environment for statistical computing version 3.5.2, with the package "lme4" version 1.1-19.

## Supplementary Information

Supplementary Information 1.

## Data Availability

The datasets generated during and/or analyzed during the current study are available from the corresponding author on reasonable request.

## References

[1] Sawyer M, Ratain MJ (2001). Body surface area as a determinant of pharmacokinetics and drug dosing. Investig. New Drugs.

[2] Daniels SR, Kimball TR, Morrison JA, Khoury P, Meyer RA (1995). Indexing left ventricular mass to account for differences in body size in children and adolescents without cardiovascular disease. Am. J. Cardiol..

[3] Hallynck TH (1981). Should clearance be normalised to body surface or to lean body mass?. Br. J. Clin. Pharmacol..

[4] Pinkel D (1958). The use of body surface area as a criterion of drug dosage in cancer chemotherapy. Cancer Res..

[5] Freireich EJ, Gehan EA, Rall DP, Schmidt LH, Skipper HE (1966). Quantitative comparison of toxicity of anticancer agents in mouse, rat, hamster, dog, monkey, and man. Cancer Chemother. Rep..

[6] Cosolo WC (1994). Lean body mass, body surface area and epirubicin kinetics. Anticancer Drugs.

[7] Du Bois D, Du Bois EF (1989). A formula to estimate the approximate surface area if height and weight be known. 1916. Nutr. Burbank Los Angels City Calif..

[8] Redlarski G, Palkowski A, Krawczuk M (2016). Body surface area formulae: an alarming ambiguity. Sci. Rep..

[9] Livingston EH, Lee S (2001). Body surface area prediction in normal-weight and obese patients. Am. J. Physiol. Endocrinol. Metab..

[10] May M, Schindler C, Engeli S (2020). Modern pharmacological treatment of obese patients. Ther. Adv. Endocrinol. Metab..

[11] Pai MP, Paloucek FP (2000). The origin of the ‘ideal’ body weight equations. Ann. Pharmacother..

[12] Griggs JJ (2012). Appropriate chemotherapy dosing for obese adult patients with cancer: American Society of Clinical Oncology clinical practice guideline. J. Clin. Oncol..

[13] Yu C-Y, Lo Y-H, Chiou W-K (2003). The 3D scanner for measuring body surface area: a simplified calculation in the Chinese adult. Appl. Ergon..

[14] Iannessi A, Beaumont H, Hebert C, Dittlot C, Falewee MN (2018). Computer tomography-based body surface area evaluation for drug dosage: quantitative radiology versus anthropomorphic evaluation. PLoS ONE.

[15] Lote H (2016). Febrile neutropenia rates according to body mass index and dose capping in women receiving chemotherapy for early breast cancer. Clin. Oncol. R. Coll. Radiol. G. B..

[16] Stocker G (2018). Clinical consequences of chemotherapy dose reduction in obese patients with stage III colon cancer: a retrospective analysis from the PETACC 3 study. Eur. J. Cancer Oxf. Engl..

[17] Hansen J (2015). The effect of weight-based chemotherapy dosing in a cohort of gynecologic oncology patients. Gynecol. Oncol..

[18] Schwartz J, Toste B, Dizon DS (2009). Chemotherapy toxicity in gynecologic cancer patients with a body surface area (BSA)>2 m2. Gynecol. Oncol..

[19] Chambers P, Daniels SH, Thompson LC, Stephens RJ (2012). Chemotherapy dose reductions in obese patients with colorectal cancer. Ann. Oncol..

[20] Georgiadis MS, Steinberg SM, Hankins LA, Ihde DC, Johnson BE (1995). Obesity and therapy-related toxicity in patients treated for small-cell lung cancer. J. Natl. Cancer Inst..

[21] Furlanetto J (2016). Higher rate of severe toxicities in obese patients receiving dose-dense (dd) chemotherapy according to unadjusted body surface area: results of the prospectively randomized GAIN study. Ann. Oncol..

[22] Bouleftour W (2019). Obesity and chemotherapy administration: between empiric and mathematic method review. Acta Oncol. Stockh. Swed..

