# Supplementary material for: Body surface area capping may not improve cytotoxic drugs tolerance
Source: Sci Rep. 2021 Jan 28;11:2431. doi: 10.1038/s41598-021-81792-6 (PMC7843991; doi:10.1038/s41598-021-81792-6)
Supplement: Supplementary file 1 — Supplementary Information 1. [file 41598_2021_81792_MOESM1_ESM.docx]

Title: Body surface area capping may not improve cytotoxic drugs tolerance

Wafa Bouleftour ^1,^*, Agathe Viard ^2^, Benoite Mery ^1^, Robin Chaux ^3^, Nicolas Magne ^4^, Xavier Simoens ^2^, Romain Rivoirard ^1^, and Fabien Forges ^2^

^1^ Medical Oncology department, Lucien Neuwirth Cancer Center, 42270 Saint Priest en Jarez France

^2^ Pharmacy department, Lucien Neuwirth Cancer Center, 42270 Saint-Priest en Jarez, France

^3^ Clinical research, innovation and pharmacology unit, university hospital of Saint-Étienne, 42000 Saint Etienne, France

^4^ Radiotherapy department, Lucien Neuwirth Cancer Center, 42270 Saint Priest en Jarez France

***** Correspondence:wafa.bouleftour@icloire.fr; Tel.: +00-33-477917470; Fax: +00-33-477917497

**Supplementary materials:**

Figure 1.supp material: Rate of chemotherapy prescriptions limited to 2 m² over the analyzed period.

**
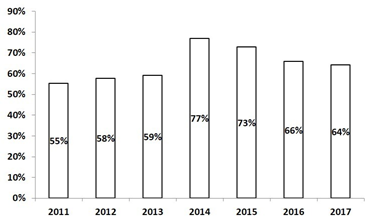
**
